# Banana fruit (Musa sp.) DNA-magnetite nanoparticles: Synthesis, characterization, and biocompatibility assays on normal and cancerous cells

**DOI:** 10.1371/journal.pone.0311927

**Published:** 2024-10-14

**Authors:** David Arregui-Almeida, Martín Coronel, Karina Analuisa, Carlos Bastidas-Caldes, Santiago Guerrero, Marbel Torres, Andrea Aluisa, Alexis Debut, Werner Brämer-Escamilla, Fernanda Pilaquinga

**Affiliations:** 1 Escuela de Ciencias Químicas, Pontificia Universidad Católica del Ecuador, Quito, Pichincha, Ecuador; 2 One Health Research Group, Universidad de las Américas, Quito, Pichincha, Ecuador; 3 Laboratorio de Ciencia de Datos Biomédicos, Universidad Internacional del Ecuador, Quito, Pichincha, Ecuador; 4 Centro de Nanociencia y Nanotecnología CENCINAT, Universidad de las Fuerzas Armadas, ESPE, Sangolquí, Pichincha, Ecuador; 5 Escuela de Ciencias Físicas y Nanotecnología, Universidad Yachay Tech, Urcuquí, Imbabura, Ecuador; National Research Centre, EGYPT

## Abstract

Magnet-mediated gene therapy has gained considerable interest from researchers as a novel alternative for treating genetic disorders, particularly through the use of superparamagnetic iron oxide nanoparticles (NPs)—such as magnetite NPs (Fe_3_O_4_NPs)—as non-viral genetic vectors. Despite their commercial availability for specific genetic transfection, such as in microglia cell lines, many potential uses remain unexplored. Still, ethical concerns surrounding the use of human DNA often impede genetic research. Hence, this study examined DNA-coated Fe_3_O_4_NPs (DNA-Fe₃O₄NPs) as potential transfection vectors for human foreskin fibroblasts (HFFs) and A549 (lung cancer) cell lines, using banana (Musa sp.) as a low-cost, and bioethically unproblematic DNA source. Following coprecipitation synthesis, DNA-Fe₃O₄NP characterization revealed a ζ-potential of 40.65 ± 4.10 mV, indicating good colloidal stability in aqueous media, as well as a superparamagnetic regime, evidenced by the absence of hysteresis in their magnetization curves. Successful DNA coating on the NPs was confirmed through infrared spectra and surface analysis results, while magnetite content was verified via characteristic X-ray diffraction peaks. Transmission electron microscopy (TEM) determined the average size of the DNA-Fe_3_O_4_NPs to be 14.69 ± 5.22 nm. TEM micrographs also showed no morphological changes in the DNA-Fe_3_O_4_NPs over a 30-day period. Confocal microscopy of HFF and A549 lung cancer cell lines incubated with fluoresceinamine-labeled DNA-Fe_3_O_4_NPs demonstrated their internalization into both the cytoplasm and nucleus. Neither uncoated Fe_3_O_4_NPs nor DNA-Fe_3_O_4_NPs showed cytotoxicity to A549 lung cancer cells at 1–50 μg/mL and 25–100 μg/mL, respectively, after 24 h. HFFs also maintained viability at 1–10 μg/mL for both NP types. In conclusion, DNA-Fe_3_O_4_NPs were successfully internalized into cells and exhibited no cytotoxicity in both healthy and cancerous cells across a range of concentrations. These NPs, capable of binding to various types of DNA and RNA, hold promise for applications in gene therapy.

## Introduction

Among contemporary medical treatments, gene therapy has emerged as a focal point of biomedical research, offering a promising way to prevent and treat diseases [1, 2]. This method involves the use of genetic material to alter the genetics underlying genome-related disorders, either by limiting or deactivating gene expression, replacing malfunctioning genes, or inserting new therapeutic ones [3, 4].

Whether DNA or RNA is administered directly into an afflicted organ (*in vivo* therapy) or into a culture of a patient’s afflicted cells that are subsequently re-transplanted (*ex vivo* therapy) [4], the success behind gene therapy depends entirely on choosing the appropriate vector for delivering genetic material [4, 5]. Among viral and non-viral vectors, the former are more frequently used despite considerable drawbacks, including potential immunogenicity, carcinogenicity, and mutagenesis [4, 6], in addition to the lack of cellular specificity, high production costs, and limited packaging capabilities [4, 7]. Hence, attention has now turned to non-viral vectors [8–11], namely nanoparticles (NPs). In the past five years, several studies on DNA-coated gold NPs have been conducted [12–16], particularly focusing on these coated NPs as functional biomedical materials (e.g., sensor/probe, tracer). Nonetheless, only a few address the delivery system by which these NPs can deliver genetic material fragments.

Magnet-mediated transfection, also known as magnetofection™, is a physical method for DNA delivery in gene therapy [4, 17, 18] that uses an external magnetic field to direct DNA-coated magnetic NPs to specific cellular targets [19, 20]. However, only a few magnetic NPs are suitable for this approach, as they must be biocompatible and have high transfection efficiency [21].

Among the magnetic NPs available for magnet-mediated transfection, biocompatible superparamagnetic iron oxide nanoparticles (SPIONs) are preferred [20, 22]. Magnetite NPs (Fe_3_O_4_NPs) are particularly favored owing to their low cost and excellent magnetic properties [23]. Under 20 nm (∼10 nm) in size, Fe_3_O_4_NPs show superparamagnetic behavior [23–25], meaning they experience rapid and strong magnetization only when exposed to an external magnetic field [23, 26].

A prominent example of using magnetic NPs as non-viral vectors in gene therapy is the novel NP formulation in the Glial-Mag reagent. This magnet-mediated transfection technology has proven successful beyond laboratory trials and is now commercially available for genetic transfection in microglia cerebral immune cell lines [27]. Nevertheless, countless cell lines associated with genetic disorders remain to be researched.

That is the case, for instance, with human foreskin fibroblast (HFF) cells. These are connective tissue-generating cells that have been positioned as ideal cell precursors candidates, given their crucial role in the tissue repair process [28]. Moreover, there are several fibroblast-related genetic disorders that do not currently respond to conventional treatments, including epidermolysis bullosa (EB). As a group of various dermal diseases, EB causes skin fragility and the rapid development of blisters with the slightest mechanical pressure [29]. Recent efforts to treat EB include stem cell, protein, and gene therapies [30, 31]. Furthermore, rare genetic disorders that currently lack effective treatments, including congenital insensitivity to pain with anhidrosis, show potential for therapeutic intervention through gene therapy [32].

Similarly, various gene therapy methods coupled with technology applying clustered regularly interspaced short palindromic repeats-associated proteins (CRISPR-Cas9) have been used to treat non-small-cell lung cancer. These methods include the activation of the immune response, insertion of self-destructive genes, blocking cancer-driving genes, or restoring genes that prevent tumor growth and promote cell death or anti-angiogenesis [33]. Within this context, SPIONs could serve as novel delivery vehicles for CRISPR-Cas9 components directly to cancerous cells.

In light of the above, SPIONs can potentially enhance the precision and efficiency of the gene editing processes, thus opening new avenues for the application of nanotechnology in rare genetic disorders and cancer gene therapy. Unfortunately, as this research falls with the realm of genetic studies, it is subject to multiple ethical concerns, particularly when using extracted DNA from human biological samples [34].

**Fig 1:**
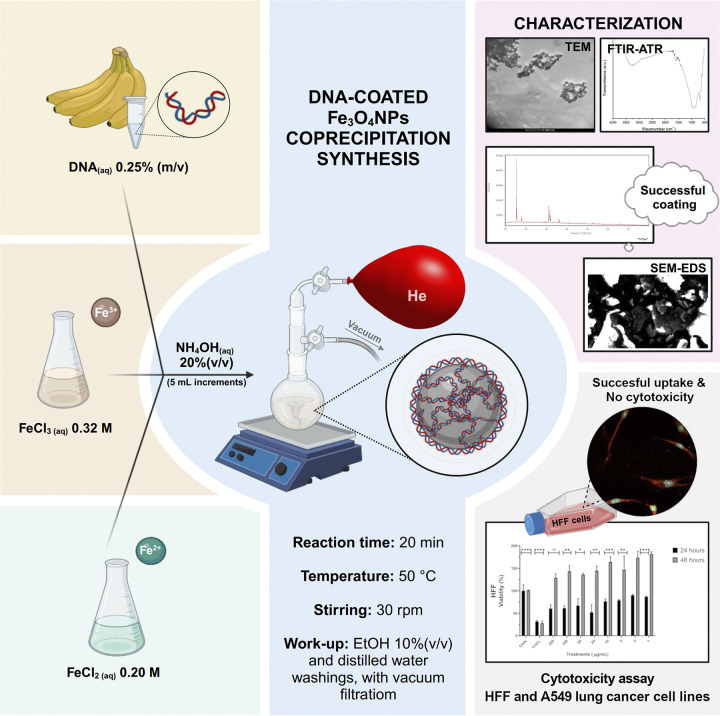
Graphical abstract. DNA-Fe_3_O_4_NPs synthesis, characterization, and cytotoxicity assay on HFF and A549 lung cancer cells.

Therefore, this study aimed to use coprecipitation to synthesize DNA-coated Fe₃O₄NPs (DNA-Fe₃O₄NPs), followed by their characterization, using bananas (Musa sp.) as an inexpensive and readily available DNA source, free from bioethical concerns (Fig 1). It also sought to assess their cytotoxic effects on previously unresearched HFF and A549 lung cancer cell lines, hence serving as a preliminary model for the use of magnetic NPs as non-viral vectors in gene therapy.

## Results and discussion

### DNA extraction

The banana sample showed a DNA concentration of 189.2 ng/μL with a 260/280 absorbance ratio of 1.82 and a 260/230 absorbance ratio of 0.70. This concentration provided the necessary amount of DNA to ensure an effective interaction with the Fe_3_O_4_NPs. Furthermore, as the 260/280 absorbance ratio was greater than 1.80, it indicated highly pure DNA with no contamination arising from fruit proteins or RNA [35, 36].

On the other hand, the 260/230 absorbance ratio suggested the presence of organic compounds, phenolics, and/or pigments specific to the fruit. Although the desirable value of this ratio is > 1.5 [37], particularly to avoid polymerase inhibition in conventional molecular biology techniques such as polymerase chain reactions (PCRs), the successful association of DNA with Fe_3_O_4_NPs is not hindered by organic compounds. Indeed, the interaction of Fe_3_O_4_NPs with certain phenolic compounds, which occur naturally as secondary metabolites in plants, may enhance their association [38].

For instance, previous studies have demonstrated that phenolic compounds derived from extracts of the plants *Vanilla planifolia* and *Cinnamomum verum* can fulfill two distinct roles for the NPs. First, they function as reducing agents precisely due to their phenolic groups. Second, they serve as coating materials through hydroxyl (OH) bonds on the surface of the Fe_3_O_4_NPs. Although the exact interactions among the system components (Fe_3_O_4_NPs, DNA, and phenolic compounds) are not entirely clear, the individual characteristics of each component offer promising advantages and implications. In particular, the environmentally friendly synthesis route for Fe_3_O_4_NPs [39], along with their potential therapeutic applications and diagnostic properties [40], underscore the need for further investigation.

### Synthesis

Over the past decades, numerous Fe_3_O_4_NPs synthesis methods have been developed [41] to control different NP variables (e.g., morphology, cost). The main advantages and disadvantages of these methods are summarized in Table 1.

**Table 1:** Comparison of magnetite nanoparticle synthesis methods.

Method	Advantages	Disadvantages	Reaction Time	Reference
**Coprecipitation**	Simple and cost-effective.	Low control over size and shape.	Minutes to hours	Syu et al. [42]
	Higher yields.			
		Broader distribution size.		
	Possible large-scale synthesis.			
**Thermal Decomposition**	Exceptional control over shape and size.	Longer reaction times. Requires high temperatures. Use of highly toxic organic solvents	Hours	Hufschmid et al. [43]
	High crystallinity.			
**Hydrothermal Method**	Good control over shape and size. Low reaction temperatures. High crystallinity.	Requires high pressurized reactors.	Days	Li et al. [44]
		Very long reaction times.		
**Sol-Gel Method**	Good control over particle size and composition.	Complex reaction procedure. Requires calcination.	Days	Takai et al. [45]
	High purity.			
**Microemulsion**	Good control over shape and size. Uniform particle size distribution.	Low yields. Use of hazardous surfactants. Scale-up difficulty.	Hours to days	Salvador et al. [46]
**Sonochemical**	Rapid reaction. Energy efficient. Uniform size and superparamagnetic distribution.	Requires ultrasonic equipment and methods.	Minutes to hours	Aliramaji, Zamanian, & Sohrabijam [47]
		Limited large-scale synthesis.		
**Electrochemical**	Simple instrumentation.	Limited nanoparticle yield.	Hours	Ramimoghadam, Samira, & Sharifah [48]
	Control over shape and size.			
		Requires precise electrolyte conditions.		
**Biogenic**	Environmentally friendly.	Very poor control over shape and size.	Days to weeks	Siddiqi et al. [49]
	Use of biological organisms.			
		Complex purification methods.		

Among the examined methods, coprecipitation was chosen for this study based on its notable advantages compared to other methods and which outweigh its own disadvantages. Given that this study required extensive characterization and biological assays, a substantial quantity of Fe_3_O_4_NPs was needed; coprecipitation effectively met this requirement [50]. Furthermore, its low cost and relatively mild reaction conditions make it a reproducible and suitable choice for alternative biomedical applications, as intended by this study [50].

The synthesized DNA-Fe_3_O_4_NPs appeared as a dark brown, powdered solid, whereas the uncoated Fe_3_O_4_NPs were darker in tone. The observed brown color probably resulted from the presence of maghemite (γ-Fe_2_O_3_), which is a common conversion product of Fe_3_O_4_ due to its relatively unstable nature [51].

### Characterization

The characterization assay results for the synthesized NPs are presented as follows: ζ-potential, Fourier transform infrared spectroscopy-attenuated total reflectance (FTIR-ATR), scanning and transmission electron microscopy (SEM and TEM, respectively), powder X-ray diffraction (XRD), magnetization, and NP stability analysis.

#### ζ-potential analysis

**Fig 2:**
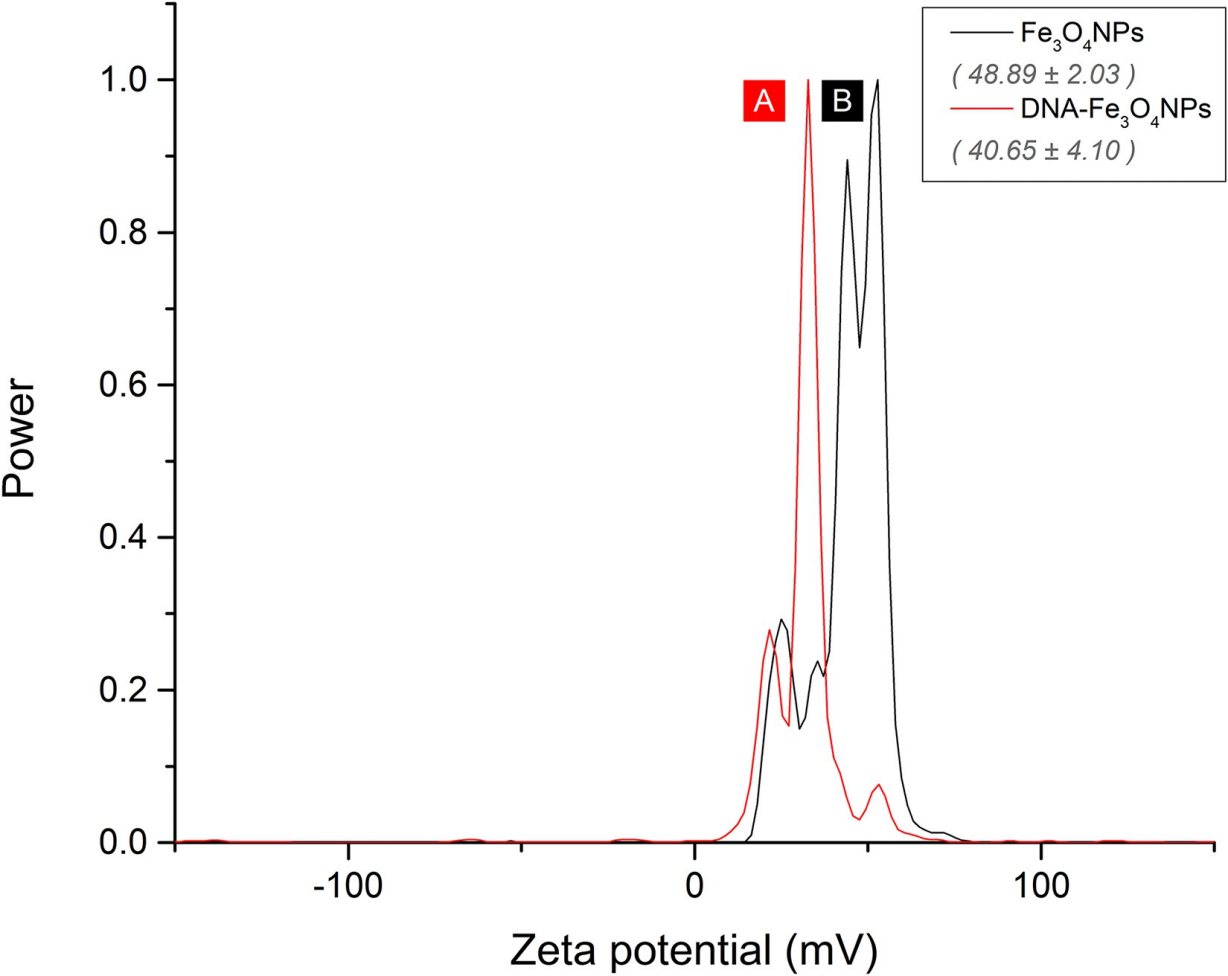
ζ-potential graph. (A) DNA-Fe_3_O_4_NPs; (B) uncoated Fe_3_O_4_NPs.

The ζ-potential results for both uncoated Fe_3_O_4_NPs and DNA-Fe_3_O_4_NPs are shown in Fig 2. The corresponding ζ-potential graphs are jointly displayed, along with their mean measurement values.

In general, the graphs exhibit a positive ζ-potential for both NP types, indicating their surfaces possess positive charges under the experimental conditions (i.e., aqueous media, pH 3). Previous studies have found that Fe_3_O_4_NPs usually carry positive charges on their surface due to the protonation of superficial hydroxyl groups under acidic conditions [52]. Although the synthesis was carried out under alkaline conditions provided by aqueous NH_3_ in the current study, it is important to consider that the NPs precipitation with NH_3_ results in the formation of the weak acid NH_4_^+^ (i.e., NH_4_Cl synthesis subproduct), which ultimately lowers the pH of the reaction medium.

The mean ζ-potential measurements for Fe_3_O_4_NPs and DNA-Fe_3_O_4_NPs were 48.89 ± 2.03 and 40.65 ± 4.10 mV, respectively. These values suggest the synthesized NPs were heavily charged on their surfaces, resulting in strong electrostatic repulsion among them. According to the literature, NPs with an absolute ζ-potential over 30 mV exhibit very good colloidal stability [53].

Nevertheless, as shown by the mean values in Fig 2, the ζ-potential of DNA-Fe_3_O_4_NPs is approximately 7 mV lower than that of Fe_3_O_4_NPs, likely due to the DNA coating. Since DNA is a negatively charged molecule, it is possible its electrostatic interaction with the positively charged surface of Fe_3_O_4_NPs neutralizes some of the original charge on the bare Fe_3_O_4_NPs. This effect has been reported when positively charged Fe_3_O_4_NPs interact with anionic organic molecules such as citric acid or poly(acrylic acid) [52], as well as with tannic acid [54].

#### FTIR-ATR analysis

**Fig 3:**
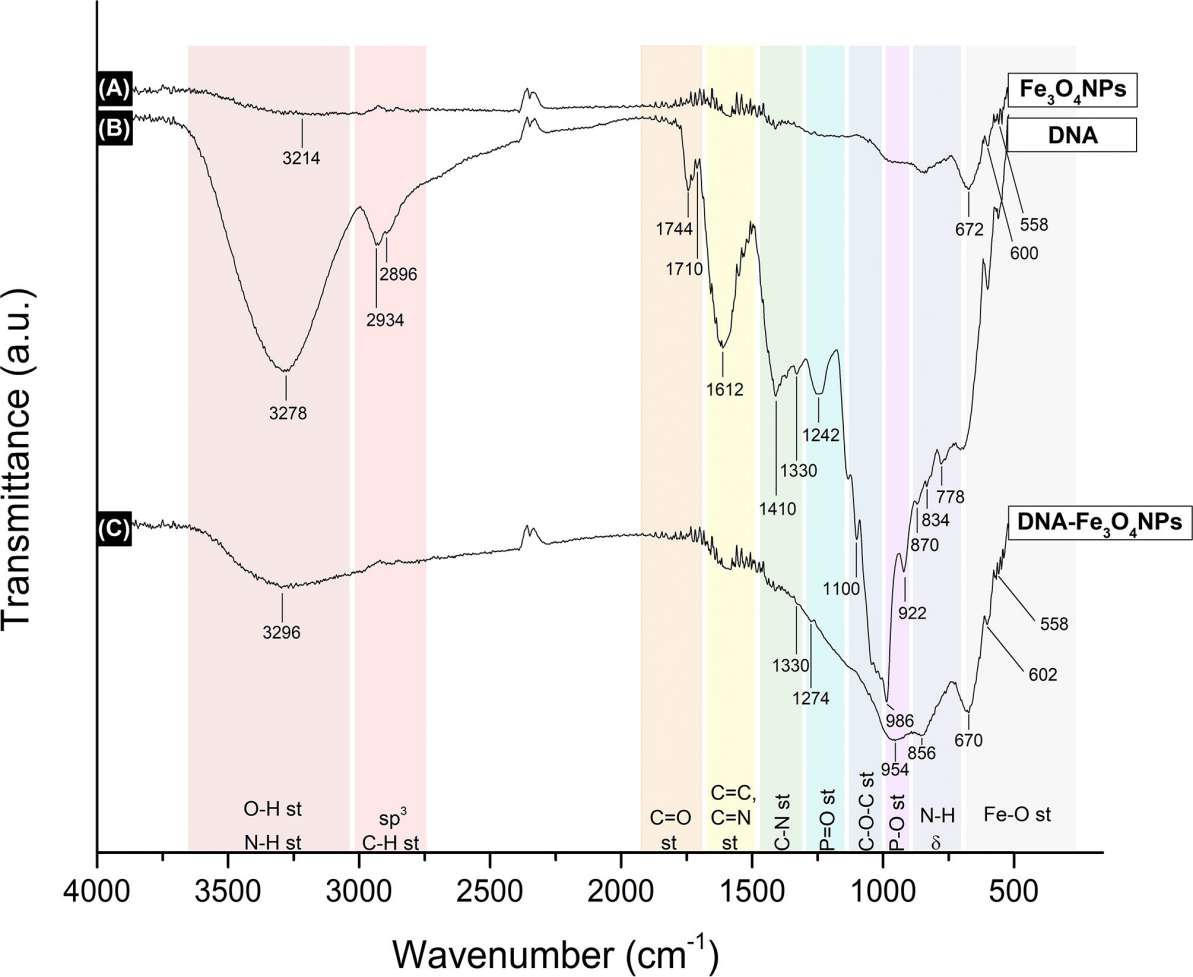
Comparative FTIR-ATR spectra. (A) uncoated Fe_3_O_4_NPs; (B) extracted DNA; (C) DNA-Fe_3_O_4_NPs.

The FTIR-ATR spectrum of the DNA-Fe_3_O_4_NPs, along with those for the uncoated Fe_3_O_4_NPs and extracted banana DNA, are jointly presented in Fig 3.

For the uncoated Fe_3_O_4_NPs, four absorption bands were identified (Fig 3A). Those found in the fingerprint region, at 672, 600, and 558 cm^-1^, are attributable to the magnetite’s Fe-O bond stretching vibration, as per similar reports [55–61]. Additionally, the absorption band at 3214 cm^-1^ describes the O-H bond stretching vibrations, likely from the hydroxyl groups found on the uncoated Fe_3_O_4_NP surface [58, 60].

In Fig 3B, 14 bands are observed, all corresponding to bond vibrations in the DNA’s characteristic functional groups.

The absorption band at 3278 cm^-1^ is within the range associated with the stretching of the N-H bond in imides and secondary amides [62], which are functional groups present in the five-membered rings of DNA’s thymine and cytosine, respectively. In particular, the existence of amides from thymine and cytosine is further supported by the presence of the N-H deformation (δ) vibration band at 870, 834, and 778 cm^-1^ [62]. The band at 3278 cm^-1^ also corresponds to the absorption band for the O-H bond stretching vibration, found in the deoxyribose hydroxyl groups [62].

The bands observed at 2934 and 2896 cm^-1^ denote the presence of sp^3^ C-H bonds, mainly found throughout the DNA’s deoxyribose. The presence of this sugar is confirmed by the 1100 cm^-1^ band, indicating a C-O-C stretching vibration [62], common to cyclic ether moiety. Furthermore, bands in the spectral regions ranging from 1575–1300 or 1500–1250 cm^-1^ can be attributed to the nitrogenous base-sugar vibrations [63–67], specifically to the torsion of the glycosidic bond between the two moieties [64].

The bands in the 1744 and 1710 cm^-1^ regions indicate the stretching vibration of carbonyl groups in guanine, thymine, and the secondary amides of cytosine [62]. Jointly, the two bands can also account for the imide moiety in the five-membered ring in the thymine base [62]. Additionally, the presence of C-N bonds is indicated by the 1612, 1410, and 1330 cm^-1^ bands; the former band corresponds to C = N bond stretching vibrations and the latter two to the C-N bond [62]. In both cases, these bonds are found throughout the nitrogenous bases, as expected from the 1800–1500 cm^-1^ range, which typically describes the ring vibrational modes of said bases [64, 67, 68].

The remaining bands at 1274, 986, and 922 indicate vibrational modes for the DNA’s phosphate group. The first band is attributable to the P = O bond stretching in said group, while the second and third bands correspond to the P-O bond stretching, specifically that occurring in the sugar–phosphate bond. Jointly, both bands support the phosphate moieties in the DNA’s backbone, as shown in previous studies [64, 65, 67, 69].

Finally, in Fig 3C, which shows the DNA-Fe_3_O_4_NPs spectrum, characteristic bands from Fig 3A and 3B can be identified jointly, suggesting the Fe_3_O_4_NPs are coated with DNA. Although some of the most prominent bands in the DNA spectrum are no longer identifiable in the DNA-Fe_3_O_4_NPs spectrum, the bands corresponding to the C-N st, P = O st, and P-O st and N-H δ can still indicate the coating presence of the DNA.

Nevertheless, the absence of the remaining characteristic bands, especially those in the 1800–1500 cm^-1^ range, may in fact indicate the successful coating of the NPs, as new interactions between the DNA and Fe_3_O_4_NPs likely occurred after coating. It has been reported that bands in the 1800–1500 cm^-1^ range are prone to changes upon the pairing and/or stacking interactions of the DNA bases [63, 68], which, in this case, may have occurred after such bases interacted with the Fe_3_O_4_NPs’ surface hydroxyl groups during coating.

#### SEM-energy dispersive X-ray spectroscopy and TEM analysis

**Fig 4:**
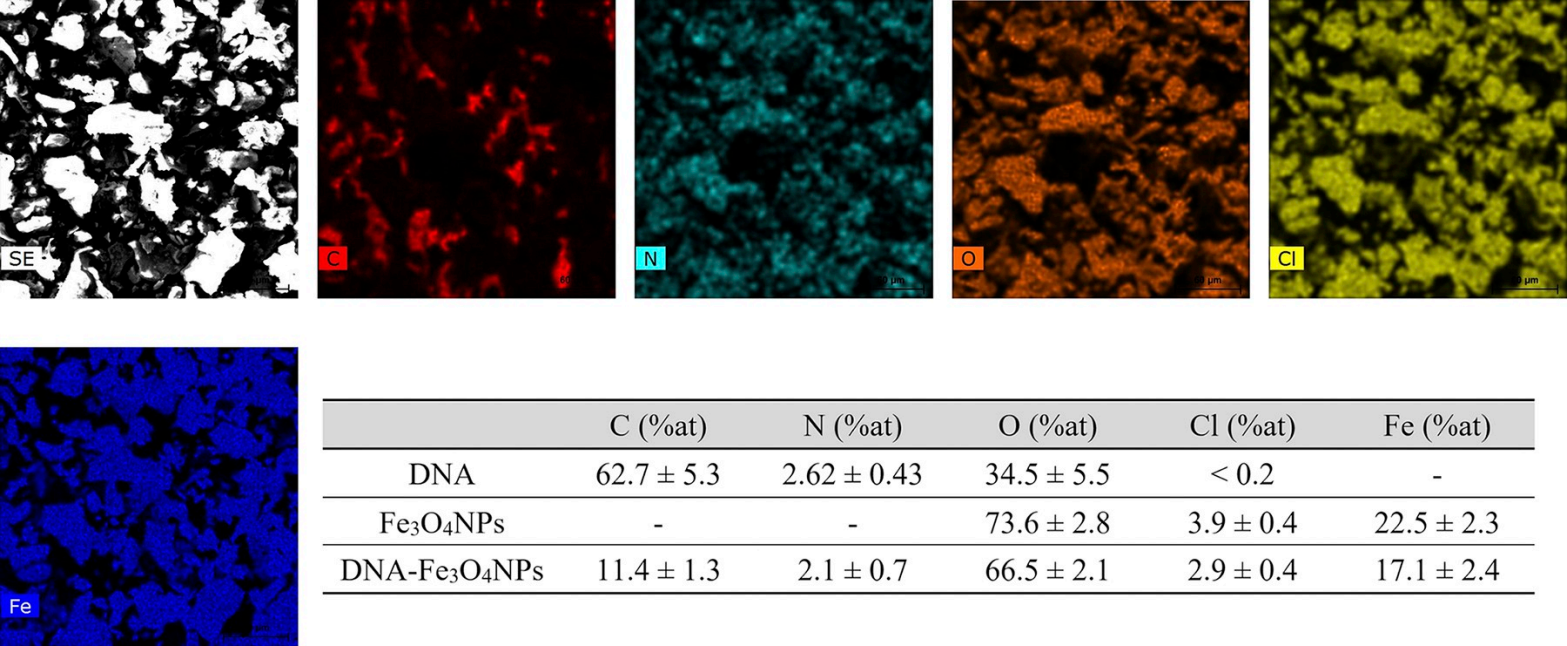
DNA-Fe_3_O_4_NP SEM-EDS mapping.

As shown in Fig 4, the energy dispersive X-ray spectroscopy (EDS) analysis of a selected area of the DNA-Fe_3_O_4_NPs confirmed the presence of O and Fe, elements found in magnetite, as well as nitrogenous base and/or sugar elements (C, N, O) present in DNA. The corresponding EDS spectrum readily identified the presence of O and Fe, with the iron emission energy observed at 6.405 keV, which corresponds to the Kα1 peak. The concurrent presence of all the aforementioned elements in the synthesized NPs indicates the successful coating of DNA on Fe_3_O_4_NPs, which is also supported by the FTIR analysis.

The presence of Cl on the DNA-Fe_3_O_4_NPs (less than 5%) is most likely due to unreacted iron (II) and (III) chloride residues (i.e., magnetite precursors). Presumably, these were trapped in the NPs’ crystalline lattice during the coprecipitation synthesis and could not be removed during the reaction work-up (i.e., chloride precipitation). This issue has been previously described as the result of NH_4_Cl (a synthesis subproduct) being trapped on the NPs despite water washings [70]. Other studies have noted that chloride ions can be absorbed onto the Fe_3_O_4_NP surfaces through the replacement of Fe-OH groups with Fe-Cl [71, 72].

Intuitively, it can be assumed Fe_3_O_4_NPs acquire a negative charge upon chloride adsorption, which could potentially affect DNA coating efficiency. However, it is important to consider that both Fe-OH and Fe-Cl groups are typically stabilized by positive counterions (e.g., Fe-Cl^-^ X^+^) [71, 72]. These positive counterions may facilitate the coating process by attracting negatively charged DNA molecules through electrostatic interactions. Based on the ζ-potential and FTIR results, it can be argued that DNA coating was successfully achieved despite the residual chloride content on the Fe_3_O_4_NPs.

**Fig 5:**
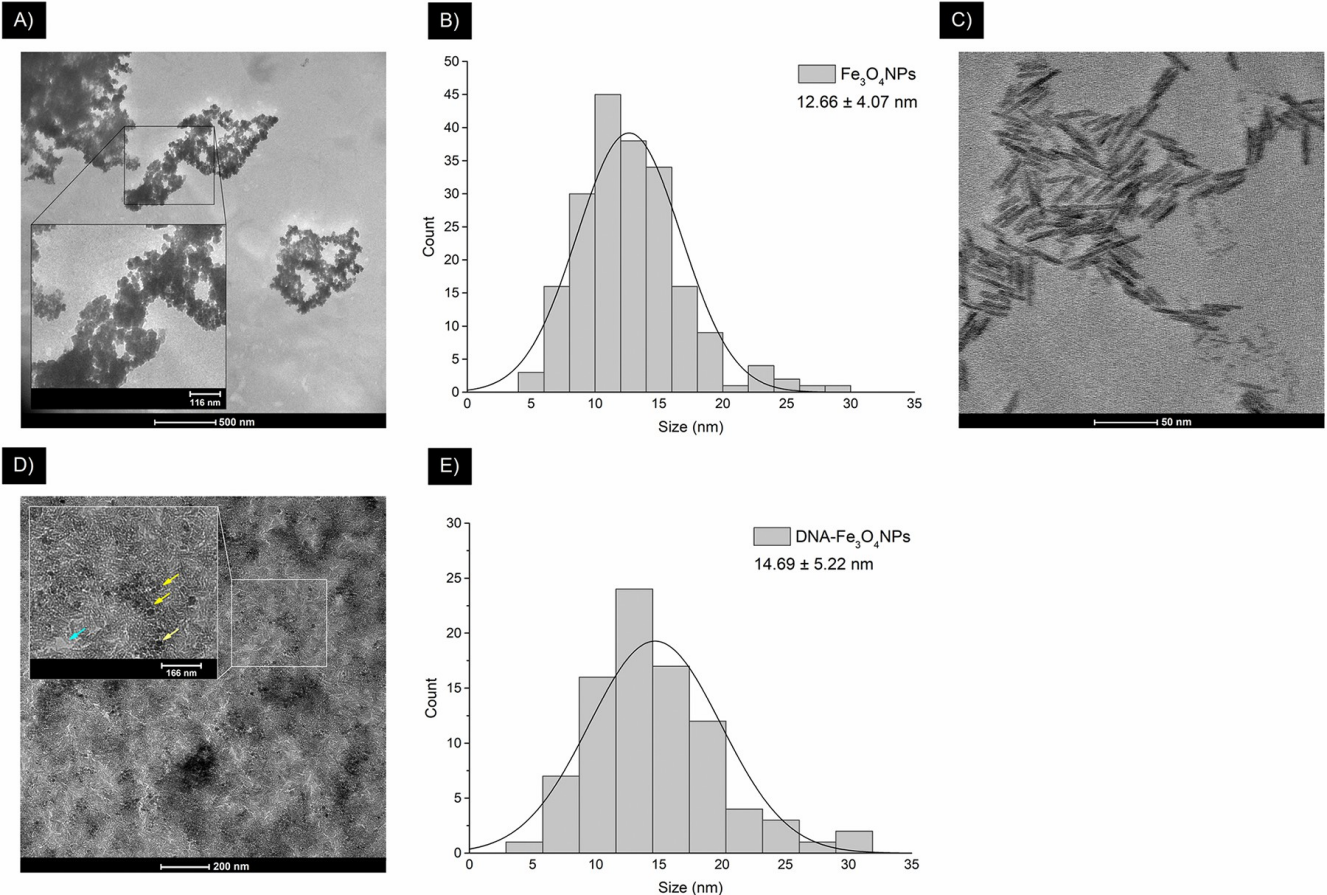
TEM micrographs for synthesized NPs and extracted DNA. (A) uncoated Fe_3_O_4_NPs; (B) size distribution histogram of uncoated Fe_3_O_4_NPs; (C) DNA; (D) DNA-Fe_3_O_4_NPs (nuclei, shown by yellow arrows; coating, shown by sky blue arrows); (E) size distribution histogram of DNA-Fe_3_O_4_NPs.

Regarding the TEM analysis, micrographs for the synthesized NPs and extracted DNA are shown in Fig 5A–5E.

As seen in Fig 5A, the majority of the Fe_3_O_4_NPs show aggregation; nevertheless, a quasi-spherical shape can be readily identified. This morphology is expected considering the chosen synthetic method and reaction conditions. As reported in recent studies, this is the most commonly observed shape when synthesizing Fe_3_O_4_NPs using coprecipitation [73–75]. Moreover, using ammonium hydroxide (i.e., aqueous NH_3_) as the precipitating agent [76] and maintaining inert reaction conditions [77], as in the current study, also facilitate a quasi-spherical shape.

Regarding size, Fig 5B shows a unimodal Gaussian-like size distribution and an average NP nucleus size of 12.66 ± 4.07 nm, which is well within the 1–100 nm nanoscale range; the successful synthesis of nanoscale particles is thus confirmed [78]. Moreover, this size also satisfies the 20-nm-or-below requirement for NPs’ biomedical use [79], which is the size conducive to superparamagnetic properties [23, 25].

As shown in Fig 5C, the solid extracted DNA used for coating forms rod-like structures approximately 50 nm in length. This conformation likely results from the spontaneous aggregation of DNA strands. Such behavior has been reported as a direct effect of removing diffusely bound positive counterions from the DNA [80]. These counterions, commonly found in DNA extracting solutions, are indirectly added to DNA during its isolation and purification process. Nonetheless, when these diffusely bound counterions are scarce and their charge-screening effects are reduced—such as during the current study’s DNA drying process before TEM analysis—condensed counterions layers from neighboring DNA molecule may interact, leading to subsequent aggregation [80].

The aforementioned DNA structures are also visible in Fig 5D. This micrograph shows quasi-spherical Fe_3_O_4_NPs nuclei, shown by yellow arrows, amidst a background of rod-like DNA aggregates, shown by sky blue arrows (i.e., coating). Similar to the uncoated Fe_3_O_4_NPs, a unimodal Gaussian-like size distribution was observed for DNA-Fe_3_O_4_NPs, with an average nucleus size of 14.69 ± 5.22 nm. Given that the Fe_3_O_4_NPs maintained the same quasi-spherical shape as the uncoated Fe_3_O_4_NPs, it reasonable to conclude that the addition of DNA for coating did not alter NP shape. Although to the authors’ knowledge, no reports on Fe_3_O_4_NPs have specified that they maintain their spherical shape after DNA coating, similar behavior has been observed with DNA-coated gold NPs [12–16]. Nevertheless, when Fe_3_O_4_NPs are coated with organic molecules (e.g., O-phosphoryl ethanolamine or D,L-mandelic acid), they retain their spherical shape [81].

#### XRD analysis

**Fig 6:**
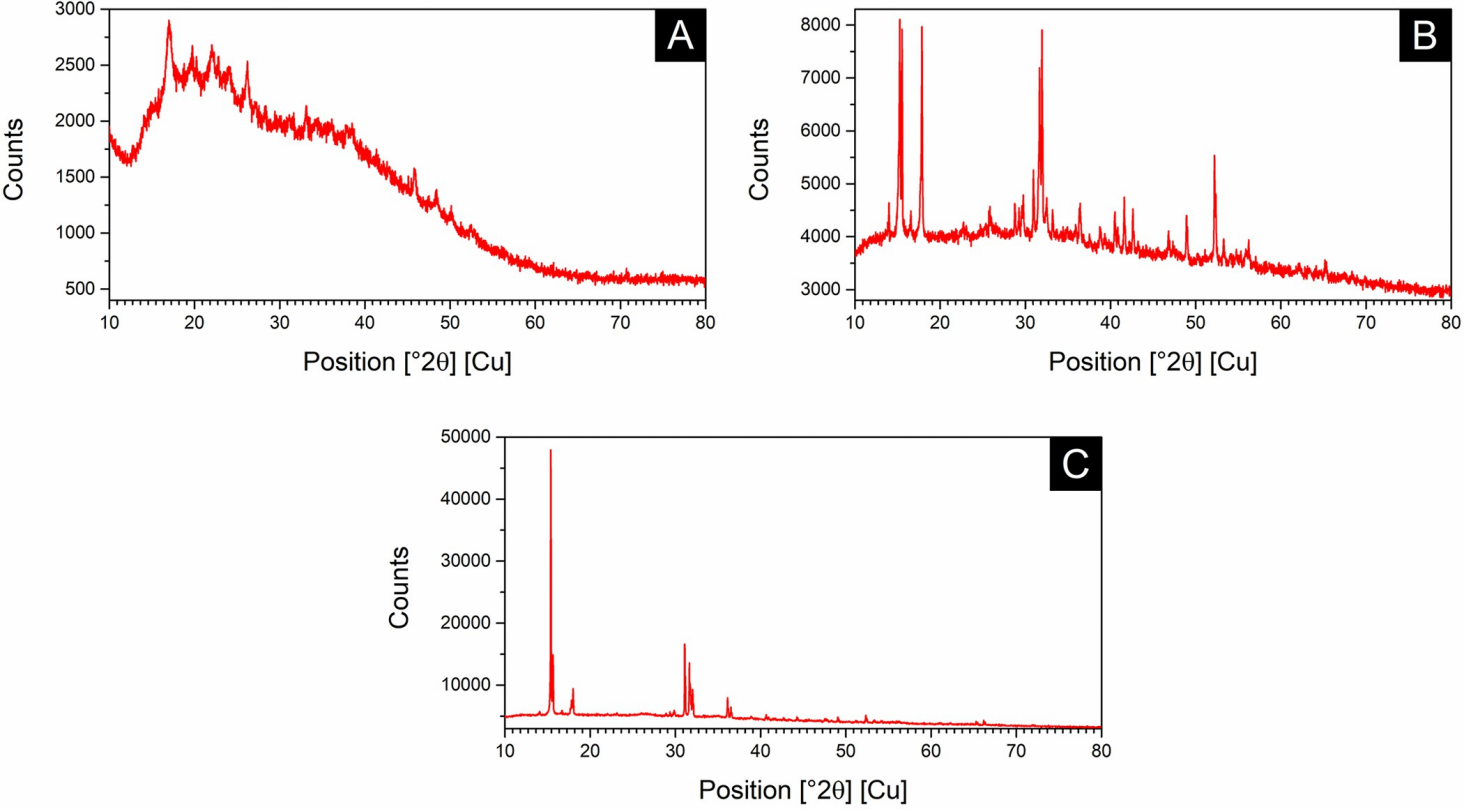
XRD diffractograms for synthesized NPs and extracted DNA. (A) DNA; (B) DNA- Fe_3_ONPs; (C) Fe_3_ONPs.

The XRD diffractograms for the extracted DNA, DNA-Fe_3_O_4_NPs, and uncoated Fe_3_O_4_NPs are respectively shown in Fig 6A–6C.

In Fig 6A, a high level of amorphous phase is indicated by a hump at a 2θ° angle around 20°. This primarily corresponds to the DNA structure and additional organic compounds potentially associated with it, as previously described by Sakar and Mandal [82]. Fig 6B and 6C show the XRD patterns of DNA-Fe_3_O_4_NPs and Fe_3_O_4_NPs, respectively. Both patterns illustrate the characteristic diffraction peaks of the magnetite phase at 35.4° [1 3 1] and 56.8° [1 5 1], corresponding to a cubic Fd-3m space group. The peaks at 26.7° and 39.2° are likely associated with maghemite. These results are consistent with previous findings [83]. The crystallite size (D) was estimated to be 15.9 nm, which is slightly larger than the TEM-determined size of approximately 10 nm.

#### Magnetization analysis

**Fig 7:**
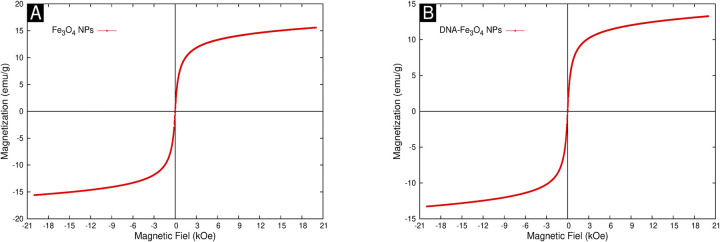
Magnetization curves for synthesized NPs. (A) Fe_3_O_4_NPs; (B) DNA-Fe_3_O_4_NPs.

Fig 7A and 7B show the magnetization behavior under an applied field for both Fe_3_O_4_NPs and DNA-Fe_3_O_4_NPs, respectively. The absence of hysteresis in both magnetization curves indicates a superparamagnetic regime for the synthesized NPs. Superparamagnetism at room temperature is usually expected in Fe_3_O_4_NPs with sizes below 20 nm [84], this being consistent with the NP sizes determined by TEM and calculations using the Scherrer equation from XRD data. However, the DNA-Fe_3_O_4_NP curve shows a 14% decrease in maximal magnetization, possibly due to the DNA coating. This phenomenon has been reported when Fe_3_O_4_NPs are coated with an organic material [81, 85, 86]. The maximal magnetization value for DNA-Fe_3_O_4_NPs was 15.6 emu/g, which is less than half the value reported in other studies [84]. This reduction is attributed to the low crystallinity of the synthesized NPs.

#### NP stability analysis

Considering the aforementioned ζ-potential analysis, no significant change in the hydrodynamic diameter of the synthesized DNA-Fe_3_O_4_NPs was expected. Thus, their stability in aqueous solution was initially assessed though dynamic light scattering (DLS) measurements. On the day of synthesis (i.e., day 1), an average value of 1 μm was found for the DNA-Fe_3_O_4_NPs. This value remained consistent 30 days later. However, it is important to note that DLS results are merely suggestive because, although all the NPs are coated with an organic molecule (i.e., DNA), they are neither spherical nor homogeneous. This explains the difference in diameter compared to the size measured via TEM.

**Fig 8:**
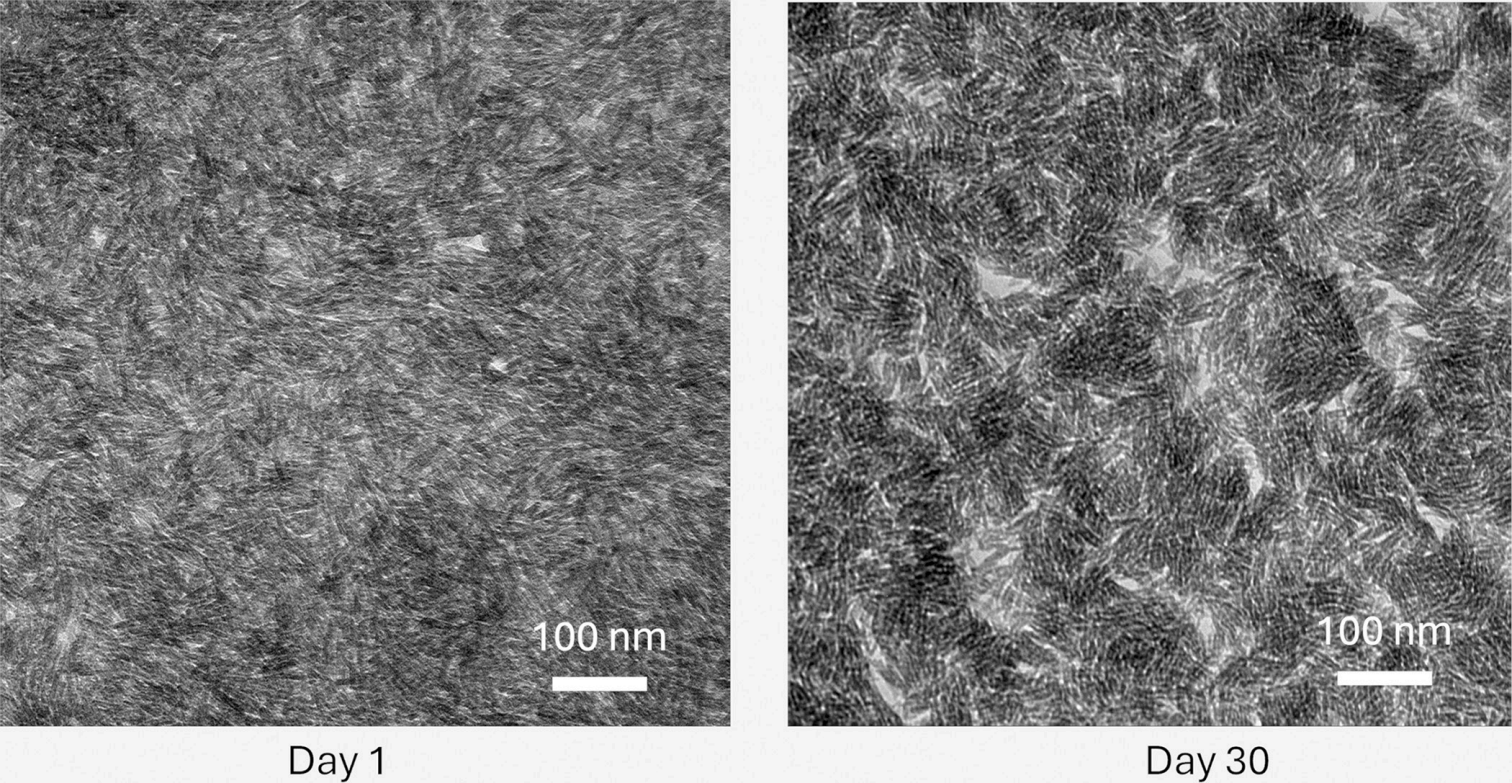
TEM micrographs for synthesized DNA-Fe_3_O_4_NPs on day one of synthesis and after 30 days.

A better assessment of stability can be achieved by directly measuring NP size using TEM images. Fig 8 shows the recorded TEM micrographs for days 1 and 30, indicating no apparent morphological changes. The measured average size for the DNA-Fe_3_O_4_NPs was 14.69 ± 5.22 nm and 10.80 ± 2.90 nm on days 1 and 30, respectively.

### Cellular assays

#### Cytotoxicity evaluation

**Fig 9:**
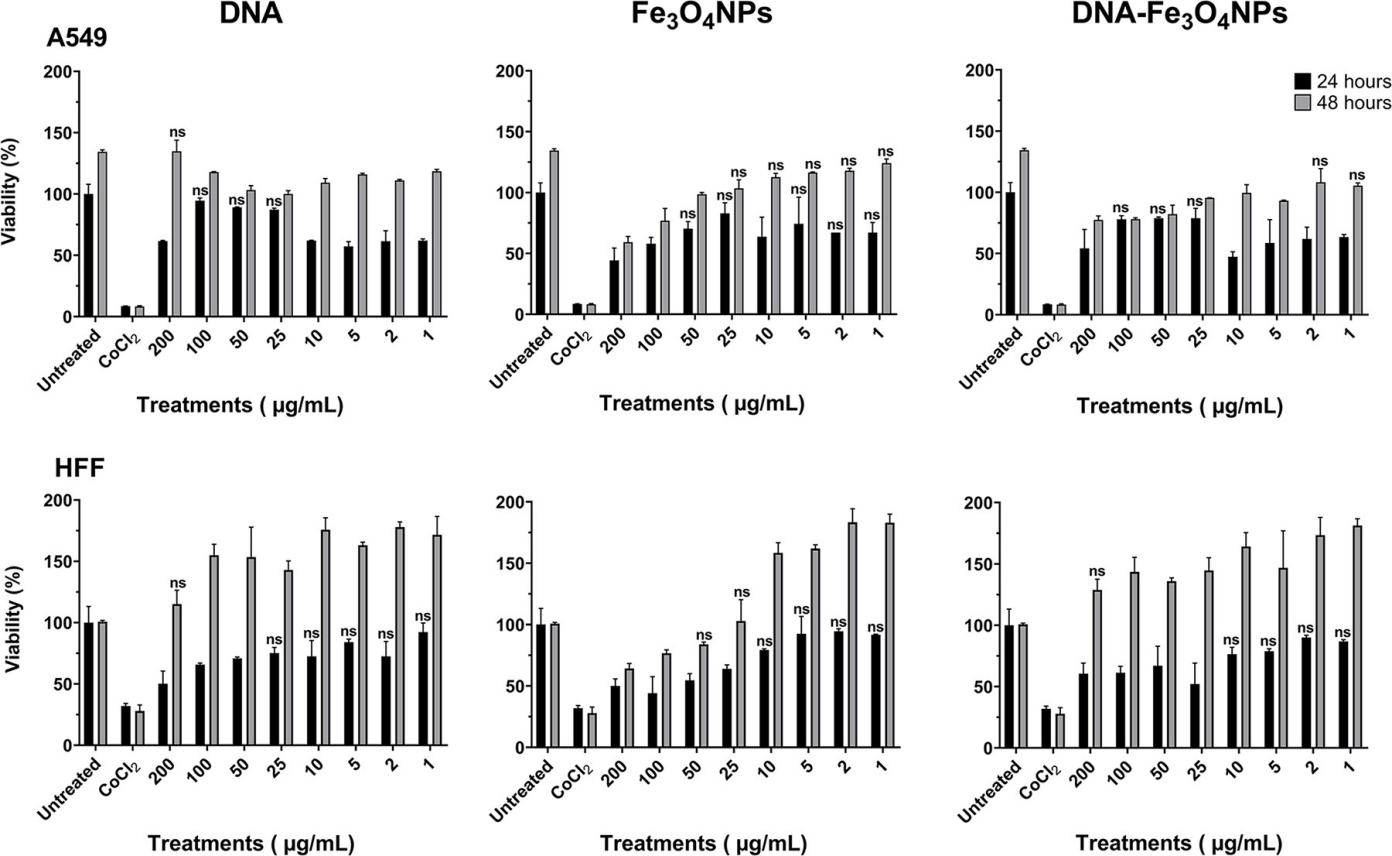
DNA-Fe_3_O_4_NP and Fe_3_O_4_NP biocompatibility with HFF and A549 cell lines. Cytotoxic effects of uncoated Fe_3_O_4_NPs and DNA-Fe_3_O_4_NPs on HFF and A549 lung cancer cells. Cell viability following exposure to different concentrations of uncoated Fe_3_O_4_NPs and DNA-Fe_3_O_4_NPs at various concentrations (200–1 μg/mL) for 24 and 48 h. Culture media (untreated) and 400 μM of CoCl_2_ were used as negative and positive controls, respectively. All comparisons between the treatments and the negative control (untreated condition) were statistically significant (*p* ≤ 0.01, two-way ANOVA followed by Dunnett’s multiple comparison test), except when marked “ns” (not significant).

After evaluating the cellular uptake of the synthesized DNA-Fe_3_O_4_NPs, their cytotoxic effects were also assessed. For A549 cells exposed to uncoated-Fe_3_O_4_NPs, no cytotoxic effects were observed at concentrations of 50, 25, 5, 2, and 1 μg/mL after 24 h (Fig 9; Dunnett’s test, not significant). Similarly, after DNA-Fe_3_O_4_NP exposure, no cytotoxicity was indicated at concentrations of 100, 50, and 25 μg/mL after 24 h (Fig 9; Dunnett’s test, not significant). After 48 hours, the viability of A549 cells remained stable at these concentrations (Fig 9). For HFF cells, exposure to 10–1 μg/mL of uncoated and DNA-Fe_3_O_4_NPs did not result in increased cell toxicity (Fig 9; Dunnett’s test, not significant). However, exposure to DNA-Fe_3_O_4_NPs at concentrations of 200–25 μg/mL led to a decrease in cell viability by approximately 48% after 24 h. Nevertheless, after 48 h, cell viability was completely restored at all concentrations (Fig 9).

#### Confocal microscopy analysis

**Fig 10:**
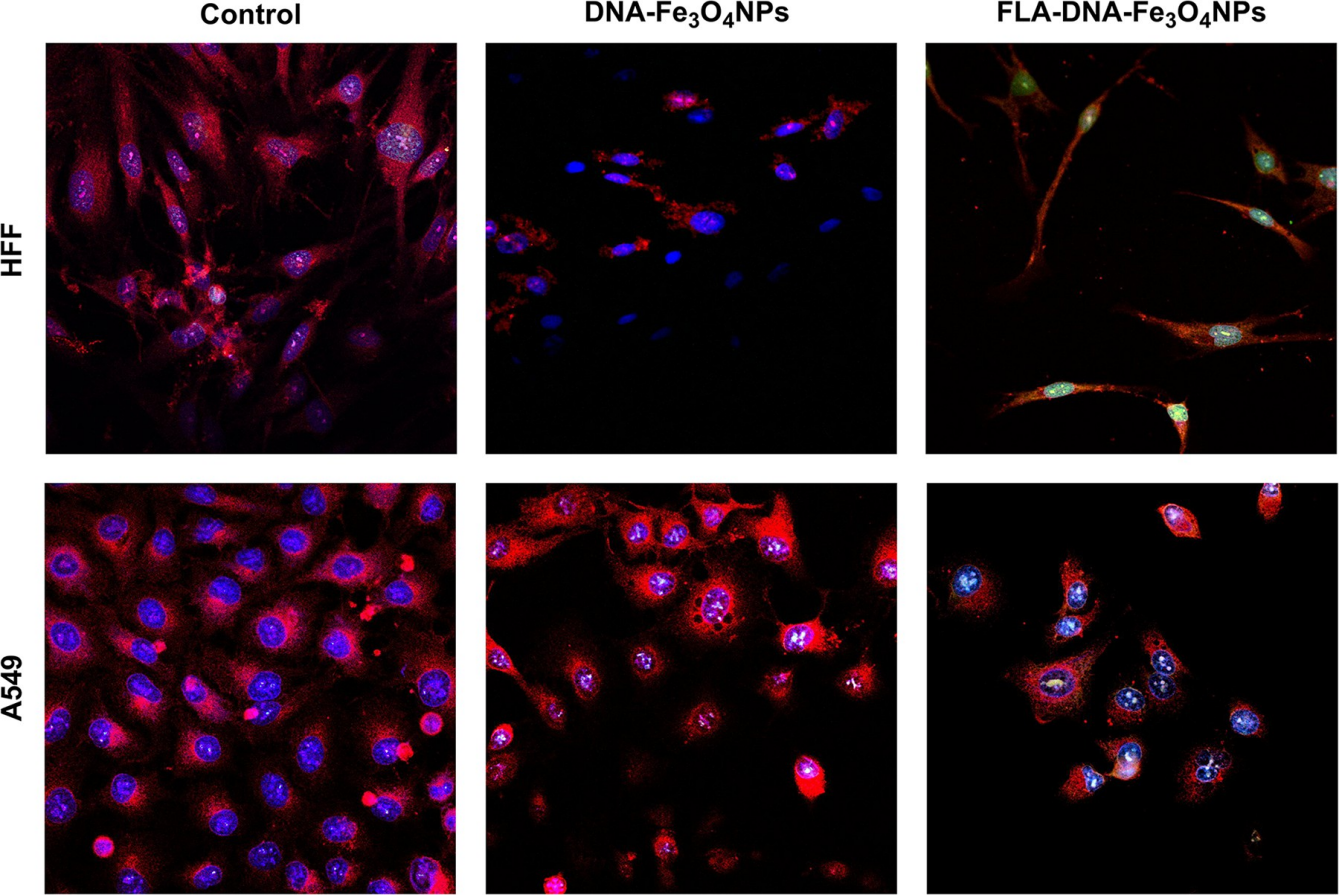
DNA-Fe_3_O_4_NP and Fe_3_O_4_NP internalization on HFF and A549 cell lines. Confocal microscopy of HFF and A549 cell lines after incubation with DNA-Fe_3_O_4_NPs and FLA-DNA-Fe_3_O_4_NPs.

To assess the potential applicability of DNA-Fe_3_O_4_NPs as genetic vectors, cellular uptake and cytotoxicity assays were performed on HFF and A549 cell lines. Confocal microscopy was used to monitor NP cellular uptake, following the approach used by other studies [87–89]. To aid visualization of NPs, fluoresceinamine (FLA)-labeled DNA-Fe_3_O_4_NPs (FLA-DNA-Fe_3_O_4_NPs) were also used at a treatment concentration of 100 μg/mL. The results for both FLA-labeled and bare DNA-Fe_3_O_4_NP in the cellular uptake and cytotoxicity assays are shown in Fig 10. After 24 h of incubation, DNA-Fe_3_O_4_NPs were internalized into both the cytoplasm and nucleus of the HFF and A549 cells. This result was confirmed by the characteristic green fluorescence of FLA-DNA-Fe_3_O_4_NPs, indicating the presence of NPs within these cellular structures.

Overall, DNA-Fe_3_O_4_NPs internalized into the cells and exhibited no cytotoxicity in both healthy and cancerous cells across a range of concentrations. These results are consistent with previous studies using other types of SPIONs [90, 91]. For example, two studies showed that mesenchymal stem cell (MSC) viability and apoptosis did not change after exposure to SPIONs [90, 91]. Similarly, Li *et al*. [92] demonstrated that SPIONs coated with heparin did not present any effect on cultured MSCs.

## Future implications

The physicochemical characteristics exhibited by the synthesized DNA-Fe₃O₄NPs have important implications for prospective biomedical applications. Despite the focus on alternative delivery systems such as DNA-coated gold NPs, their use has been limited by low efficacy arising from the DNA/vector complexes’ short-lived presence in the bloodstream [93] and poor colloidal stability in physiological environments [94]. Instead, DNA-Fe₃O₄NPs offer a cost-effective delivery system alternative for difficult-to-access target cells, particularly because their highly magnetic properties enable precise motion and direction via external devices.

Interestingly, emerging strategies for gene editing and therapy utilizing proteins associated with CRISPR-Cas9 technology are actively seeking efficient delivery systems. In this regard, magnetite NPs have already shown promising results [18, 95, 96]. Moreover, the use of magnetic NPs, including magnetite, has yielded encouraging results in *in vivo* genetic therapy assays [97, 98] and for potential *ex vivo* analogues [99]. Thus, DNA-Fe₃O₄NPs could serve as an optimal transfection method in genome-editing systems, applicable not only for *in vivo* and *ex vivo* therapies but also for fundamental research.

Nonetheless, the true success behind all potential applications lies in the biocompatible nature of the DNA-Fe₃O₄NPs, as shown by the cytotoxicity assay. The organic DNA coating combined with magnetite’s nontoxic nature guarantees the suitability of these NPs in *in vivo* systems. Without this biocompatibility, numerous biomedical applications would be impossible.

## Conclusions

DNA-Fe_3_O_4_NPs, with a quasi-spherical shape averaging 14.69 ± 5.22 nm in size, were synthesized via coprecipitation and using banana (Musa sp.) as an inexpensive, readily available, and ethically unobjectionable DNA source. These NPs exhibited a ζ-potential of 40.65 ± 4.10 mV, indicating good colloidal stability in aqueous media, and displayed superparamagnetism, as evidenced by the absence of hysteresis in their magnetization curves. Infrared spectra of the DNA-Fe_3_O_4_NPs revealed distinctive absorption bands corresponding to Fe-O bond stretching and DNA functional groups’ vibrations. SEM-EDS analysis confirmed the presence of C, O, N, and Fe, indicating successful DNA coating on the NPs. XRD results further confirmed magnetite content on the synthesized NPs through characteristic diffraction peaks. Moreover, TEM micrographs showed no morphological changes in the DNA-Fe_3_O_4_NPs over a 30-day period.

Confocal microscopy showed that DNA-Fe_3_O_4_NPs were successfully internalized by both HFF and A549 lung cancer cells. Cytotoxicity assays indicated that neither Fe_3_O_4_NPs nor DNA-Fe_3_O_4_NPs were cytotoxic to HFF and A549 lung cancer cells, across a concentration range of 1 to 100 μg/mL after 24 and 48 h of exposure. These results are attributable to the DNA-Fe_3_O_4_NPs’ composition and coating, and they indicate their potential for biomedical applications such as gene therapy.

Further studies should focus on evaluating the performance of DNA-Fe_3_O_4_NPs as genetic vectors in both normal and cancerous cell lines, particularly when carrying specific genetic sequences or used in conjunction with CRISPR-Cas9 technology. Additionally, new coating strategies should be explored for bare Fe_3_O_4_NPs that consider the chemistry of the specific nucleic acid sequences under assay. Alternative methods for coating bare Fe_3_O_4_NPs should be assessed, particularly those that allow for reversible anchoring of genetic material on the NP surface.

## Materials and methods

All the following reagents were of analytical grade and used without further purification: iron (II) chloride tetrahydrate (FeCl_2_·4H_2_O, 98%, BDH Chemicals Ltd., Poole, England), iron (III) chloride hexahydrate (FeCl_3_·6H_2_O, 97%, Loba Chemie, Mumbai, India), potassium chromate (K_2_CrO_4_, 99.5%, Merck, Darmstadt, Germany), sodium chloride (NaCl), 10% (v/v) ethyl alcohol (EtOH), and 20% (v/v) ammonium hydroxide (NH_4_OH, aqueous NH_3_).

### Banana DNA extraction

A sample of banana (Musa sp.) was peeled and blended in TE buffer inside a sterile bag in a Stomacher® 80 Biomaster laboratory blender (Seaward, Worthing, United Kingdom) to form a paste; 1 mL of sample was then transferred to a clean 1.5 mL microtube. To obtain high-quality genomic plant DNA, the DNeasy® Plant Mini Kit (Qiagen, Germany) was used for DNA extraction, following the manufacturer’s instructions. Initially, 1 mL of banana sample was transferred to a tissue disruption tube containing a specially shaped bead and buffer to facilitate homogenization and cell lysis through shaking. Subsequently, the released genomic DNA was purified by removing PCR inhibitors using inhibitor removal solutions and was captured on a silica membrane in a centrifugation column. This process involved the elimination of cell debris, precipitated proteins, and polysaccharides through washing solutions and incubation of the sample with proteinase and RNAase. The purified DNA was then resuspended and recovered in a new 1.5 mL tube for storage at -20°C. Subsequently, 2 μL of the DNA sample was quantified using a NanoDrop spectrophotometer (Thermo Scientific, Waltham, Massachusetts, USA), assessing the concentration and purity based on absorbance ratios at 260 and 280 nm, before storing the sample at -20°C.

### DNA-Fe_3_O_4_NP coprecipitation synthesis

DNA-Fe_3_O_4_NPs were synthesized using the modified coprecipitation method reported by Pilaquinga *et al*. [100]. In a 150 mL flat-bottom flask, 50 mL of aqueous 0.32 M FeCl_3_·6H_2_O was mixed with 50 mL of aqueous 0.2 M FeCl_2_·4H_2_O and 50 mL of aqueous 0.25% (m/v) DNA solution. The resulting solution was heated at 50°C for 10 min under constant stirring (30 rpm) (magnetic stirrer, Velp® Scientifica, New York, USA). Next, 20 mL of 20% (v/v) aqueous NH_3_ was slowly added in 5 mL increments using a circular motion along the inside walls of the reaction flask. The flask was then immediately placed under an inert helium atmosphere.

After 20 min, DNA-Fe_3_O_4_NPs were vacuum-filtered (vacuum pump, BOECO, Hamburg, Germany) through MN 615 paper (Macherey-Nagel, Düren, Germany). From the remaining aqueous suspension, a 1 mL sample was filtered through a 0.22 μm PVDF microfilter (Milllipore Millex-GV, Thermo Scientific, Waltham, Massachusetts, USA) and stored at -4°C for the TEM characterization and stability analysis. Two additional 10 mL samples were stored at -4°C for the ζ-potential analysis. Prior to analysis, these samples were centrifuged, washed, and resuspended in distilled water.

For the remaining DNA-Fe_3_O_4_NPs on the filter paper, thorough rinses were performed, first with 10% (v/v) EtOH and then with distilled water. The presence of chloride ions was then qualitatively evaluated by precipitation with aqueous 5% (m/v) AgNO_3_ and using 5% (m/v) K_2_CrO_4_ as an indicator. Washings were repeated until no chloride precipitation was observed. Subsequently, the NPs were dried under infrared light (Maviju, China) for 10 days. They were then weighed (analytical balance, Mettler Toledo, New Classic, ML204, Columbus, Ohio, USA) and stored in dry conditions until needed for FTIR-ATR, SEM-EDS, XRD, magnetization, and cytotoxicity analysis.

Synthesis and sample preparation for uncoated Fe_3_O_4_NPs followed the same procedure.

### Preparation of FLA-DNA-Fe_3_O_4_NPs

A 5 mL solution of 3.6 mg/mL FLA (isomer I, Sigma-Aldrich, St. Louis, Missouri, USA) was prepared using water/acetone 3:2 mixture as the solvent. To label the DNA-Fe_3_O_4_NPs, a 5 mL aqueous suspension of the NPs was sonicated for 15 min (Branson 3510 ultrasonic bath, Brookfield, Connecticut, USA). Subsequently, 1 mL of the FLA solution was added to the 5 mL suspension and stirred for 5 h, at 30 rpm and room temperature. The acetone content was then removed by rotary evaporation. The labeled DNA-Fe_3_O_4_NPs were microfiltered through a 0.22 μm PVDF microfilter (Milllipore Millex-GV, Thermo Scientific, Waltham, Massachusetts, USA) and stored in dark conditions until use in cellular uptake assays.

### DNA-Fe_3_O_4_NP characterization

#### ζ-potential & stability analysis

The ζ-potential measurements of the DNA-Fe_3_O_4_NPs were determined using a 90 Plus Brookhaven ζ-potential analyzer (Illinois, USA). Their stability in aqueous media was assessed through weekly DLS measurements over a one-month period (i.e., 30 days) using an LB-550 DLS NP size analyzer (Horiba, Kyoto, Japan). The results were processed with OriginLab 9.0 (OriginLab Corporation, Washington, USA). Similarly, Fe_3_O_4_NPs were analyzed to obtain their respective ζ-potential and DLS measurements.

#### FTIR-ATR spectroscopy

The infrared spectrum of the DNA-Fe_3_O_4_NPs were determined using an FTIR-ATR module spectrometer (Perkin Elmer, Spectrum BX II, Waltham, Massachusetts, USA) in the 4000–520 cm^-1^ range, with 10 scans per analysis and a 4 cm^-1^ and 2 cm^-1^ spectral resolution and interval, respectively. The resulting spectrum was processed with OriginLab 9.0. The corresponding spectra for extracted DNA and uncoated Fe_3_O_4_NPs were also recorded under the same conditions.

#### SEM-EDS and TEM analysis

The elemental composition of the DNA-Fe_3_O_4_NPs was analyzed via EDS using a field emission Tescan Mira 3 scanning electron microscope (Brno-Kohoutovice, Czech Republic) equipped with a Bruker X-Flash 6–30 detector (Billerica, Massachusetts, USA) with a resolution of 123 eV at Mn Kα. Their microstructure was observed at 80 kV with TEM in an FEI-Tecnai G20 Spirit Twin transmission electron microscope (Hillsboro, Oregon, USA) equipped with an Eagle 4K HR camera and a LaB6 filament. Resulting micrographs were processed with Fiji: ImageJ software (USA) to measure the NPs’ diameter and determine their average size. Subsequently, size distribution results were analyzed with OriginLab 9.0. Under the same conditions, the SEM-EDS and TEM analysis were also carried out for the uncoated Fe_3_O_4_NPs and solid extracted DNA.

Additional TEM micrographs were recorded on the day the DNA-Fe3O4NPs were synthesized and after a 30-day period to assess their stability in terms of observable morphology and NPs size changes. DNA-Fe_3_O_4_NP sizes were determined as described previously.

#### XRD analysis

DNA-Fe_3_O_4_NPs, uncoated Fe_3_O_4_NPs, and extracted DNA were characterized using XRD to evaluate their crystalline structure. For this purpose, a Malvern Panalytical Empyrean X-ray diffractometer equipped with a copper X-ray tube (Kα radiation, λ = 1.54056 Å) was used. The XRD data were collected in the 2Θ range from 5° to 90° with a scan rate of 0.01° at 45 kV and 40 mA. All samples were prepared on zero-background support. Due to the low concentration of the magnetite NPs, a fast Fourier fit was applied to the diffractograms of the uncoated Fe_3_O_4_NPs and DNA-Fe_3_O_4_NPs for better visualization; however, raw data were used for processing. Highscore© software (Panalytical, Malvern, United Kingdom) was used for data interpretation, connected to the Crystallography Open Database. Crystallite size (D) was calculated using the Scherrer equation, with the full width at half maximum (FWHM) value obtained via the same software using a Pseudo-Voigt fit on the peak of greatest intensity. A Scherrer constant *k* = 0.89, typical for spherical geometries, was considered.

#### Magnetization analysis

The magnetic responsiveness of uncoated and DNA-Fe_3_O_4_NPs was evaluated using quantitative magnetization analysis and carried out in a VersaLab Physical Property Measurement System with a vibrating sample magnetometer measurement option (Quantum Design, USA).

### Cellular assays

Prior to conducting any cellular assays, the sterility of the synthesized NPs was evaluated to ensure no bacterial contamination. The NPs were incubated on nutrient agar plates (serving as sterility controls) at 37°C for 24 h, after which bacterial growth was assessed.

#### Cytotoxicity test

The cytotoxicity of uncoated and DNA-Fe_3_O_4_NPs was evaluated using HFFs (HFF- SCRC-1041 ATCC) and lung cancer (A549) cell lines. Prior to the cytotoxicity assay, cells were tested for mycoplasma contamination using PCRs. Briefly, cellular DNA was extracted using a commercial extraction kit (Wizard Genomic DNA, A1125, Promega, USA) and stored at -20°C until further use. PCRs were performed with the extracted DNA (templates) using *Mycoplasma* spp. GPO-3 and MGSO primers and Taq DNA polymerase (Invitrogen 10342046, Thermo Fisher, USA).

After confirming the absence of mycoplasma, the cytotoxicity assay was carried out using the MTT (3-(4,5-dimethylthiazol-2-yl)2,5-diphenyltetrazolium bromide) method to assess cell viability based on mitochondrial activity. In 96-well plates, 10,000 cells per well were seeded for each test. After cell adherence, the culture medium was removed and replaced with diluted NPs suspensions (1 mg/mL), which were prepared by diluting the NPs in culture media to concentrations ranging from 1 to 200 ug/μL. Each of these solutions was tested individually in triplicate for both 24 and 48 h experimental periods.

In each test, culture media and 400 μM of CoCl_2_ (97%, Sigma-Aldrich, Darmstadt, Germany) were used as negative and positive controls, respectively. At the end of each experimental period, the added DNA-Fe_3_O_4_NP treatments were removed, and each well was rinsed twice with sterile 1X phosphate buffered saline (PBS, Eurobio, Les Ulis, France). Next, 100 μL of FluoroBrite DMEM (Gibco™ A1896701, Thermo Scientific, Waltham, Massachusetts, USA) with 10 μL of 5 mg/mL MTT solution (Sigma-Aldrich, St. Louis, Missouri, USA) was added to each well. The 96-well plates were kept at 37°C under 5% CO_2_ for 4 h. Subsequently, 100 μL of 10% SDS solution (Invitrogen, Waltham, Massachusetts, USA) was added to dissolve the formazan crystals.

All plates were analyzed after the 24 and 48 h incubation periods, under the aforementioned conditions, using a MultiSkan Go Plate Reader (Thermo-Fischer, Waltham, Massachusetts, USA), at 570 nm. Cell viability was determined based on the absorbance of the treatments and positive control with the following equation:

$$Cell´s\;viability\;(\%)=\frac{Treatments´\;absorbance\;}{Positive\;control\;absorbance}\times 100$$

The entire procedure was repeated using extracted Musa sp. DNA and uncoated Fe_3_O_4_NPs. Statistical analysis to compare treated and untreated control groups was conducted using a two-way analysis of variance, followed by Dunnett’s multiple comparison test.

#### Confocal microscopy

From a culture of fibroblast (HFF-SCRC-1041 ATCC) and lung cancer cells (A549), 250,000 cells/well were seeded in 12-well plates with cover glasses. After 24 h of incubation at 37°C with CO_2_, 250 μL of each treatment (DNA-Fe_3_O_4_NPs and FLA-DNA-Fe_3_O_4_NPs) was added to the wells. After another 24 h, the 12-well plate was exposed to a neodymium magnet (NdFeB) at room temperature for 5 min. The cells were then fixed with absolute ethanol for 10 min at -20°C. Subsequently, they were stained with rhodamine-phalloidin (10 μg/mL) for 30 min at 37°C, followed by washing with 1X PBS. Nuclei were then stained with DAPI (100 μg/mL) for 15 min at room temperature.

The cells were observed using a confocal microscope (Olympus Fluoview FV1200) with a 60X objective lens. DAPI was visualized with a 405 nm laser (excitation: 341 nm, emission: 452 nm), and rhodamine-phalloidin with a 543 nm laser (excitation: 540 nm, emission: 565 nm). The resulting micrographs were analyzed using Fiji: ImageJ software.
